# RNA-Seq-Based Transcriptomics Study to Investigate the Genes Governing Nitrogen Use Efficiency in Indian Wheat Cultivars

**DOI:** 10.3389/fgene.2022.853910

**Published:** 2022-03-31

**Authors:** Sarabjit Kaur, M. Shamshad, Suruchi Jindal, Amandeep Kaur, Satinder Singh, Achla sharma, Satinder Kaur

**Affiliations:** ^1^ School of Agricultural Biotechnology, Punjab Agricultural University, Ludhiana, India; ^2^ Department of Plant Breeding and Genetics, Punjab Agricultural University, Ludhiana, India

**Keywords:** differential gene expression, nitrogen use efficiency, wheat, transcriptome analysis, roots

## Abstract

High NUE (nitrogen use efficiency) has great practical significance for sustainable crop production. Wheat is one of the main cultivated crops worldwide for human food and nutrition. However, wheat grain productivity is dependent upon cultivars with high NUE in addition to the application of nitrogen fertilizers. In order to understand the molecular mechanisms exhibiting a high NUE response, a comparative transcriptomics study was carried out through RNA-seq analysis to investigate the gene expression that regulates NUE, in root and shoot tissue of N-efficient (PBW677) and N-inefficient (703) cultivars under optimum and nitrogen (N) stress. Differentially expressed gene analysis revealed a total of 2,406 differentially expressed genes (DEGs) present in both the contrasting cultivars under N stress. The efficient genotype PBW677 had considerably more abundant DEGs with 1,653 (903 roots +750 shoots) compared to inefficient cultivar PBW703 with 753 (96 roots +657 shoots). Gene ontology enrichment and pathway analysis of these DEGs suggested that the two cultivars differed in terms of adaptive mechanism. Gene enrichment analysis revealed that among the upregulated and downregulated genes the overrepresented and underrepresented gene categories belonged to biological processes like DNA binding, response to abiotic stimulus, photosynthesis, carbon fixation, carbohydrate metabolic process, nitrogen compound metabolic process, nitrate transport, and translation in cultivar PBW677, while the enriched biological processes were nucleosome assembly, chromatin remodeling, DNA packaging, lipid transport, sulfur compound metabolic process, protein modifications, and protein folding and refolding in N inefficient cultivar PBW703. We found several transcription factors (MYB, WRKY, RING finger protein, zinc finger protein, transporters, NRT1, amino acid transporters, sugar), protein kinases, and genes involved in N absorption, transportation, and assimilation to be highly expressed in high NUE cultivar PBW677. In our study, we report 13 potential candidate genes which showed alternate gene expression in the two contrasting cultivars under study. These genes could serve as potential targets for future breeding programs.

## Introduction

Nitrogen is one of the essential macronutrients required for plant growth, development, and reproduction. In last few decades, extensive use of N fertilizers resulted in increased biomass and crop yield. It has been estimated that by the year 2050, the application of N fertilizer will increase to 135 Tg N (Good et al., 2004). But excessive use of N causes environmental pollution, deteriorates soil health, and also leads to a higher cost of production. Thus, increased N use efficiency in plants would not only result in higher crop yield under N-stressed conditions, but also benefit farmers via higher net profit under low input and mitigate the environment risks arising due to an excess of fertilizers. Therefore, one of the main aims in agricultural research is to increase the NUE of plants which depends on plant N-uptake efficiency, N-utilization efficiency, and on the remobilization efficiency of nitrogen from dead tissue to growing plant parts (Masclaux-Daubresse et al., 2010).

Grain production in major cereals largely depends upon the application of N fertilizers and cultivars with high NUE (Hitz et al., 2017). Grain production in crop plants could be enhanced by harnessing the genetic variation for improved NUE. Nitrogen use efficiency is strongly affected by genetic as well as environmental factors (Xu et al., 2012). The key genes involved in the pathways involved in NUE can be discovered using the power of omics. Transcriptomics or RNA-seq is one such approach which can be utilized to reveal the key genes responsible for combating N stress under N-deficit conditions using contrasting genotypes or cultivars having different NUE (Kant et al., 2011). RNA-seq via next-generation sequencing platform allows the discovery of genes playing a role in pathways affecting such phenotyping traits. Many studies are available illustrating the potential of transcriptomics to decipher the role of key genes in nitrogen-dependent pathways, for example the genotypic difference in terms of nitrogen use efficiency has been studied in various crops like rice (Vijayalakshmi et al., 2015), soybean (Hao et al., 2011), sorghum (Gelli et al., 2014), and maize (Zamboni et al., 2014).

Wheat is one of the main cultivated crops worldwide (Xu et al., 2013). To meet the needs of the world’s growing population, the grain yield of wheat must be increased at an average annual rate. However, wheat yield is frequently threatened by low-nitrogen stress. Therefore, improving the nitrogen use efficiency for increased biomass as well as increased yield of wheat under N-starvation conditions has great practical significance (Curci et al., 2017). Hence, efforts have been made to understand the molecular and physiological basis of plants grown under N stress, which will help in identification of a large number of differentially expressed genes (DEGs) through RNA sequence-based transcriptomics analysis (Ruuska et al., 2008).

In wheat, attempts have been made to understand the regulatory mechanisms involved in nitrogen metabolism and various studies have been conducted so far to discover the genes via transcriptome sequencing. The RNA-seq studies have been carried out taking various tissue samples, for example leaf tissue, roots, flag leaf, etc., from a single cultivar and differential gene expression was carried out to unravel the key genes and pathways (Meng et al., 2021; Sultana et al., 2021; Tiong et al., 2021).

We have conducted a comparative transcriptomics study using the next-generation sequencing platform Illumina HiSeq 500 using two bread wheat cultivars: PBW677 with high NUE and PBW703 with low NUE (Shamshad, 2019) and studied the whole genome gene expression profile in control as well nitrogen-deficient conditions in root and shoot tissue. This is the first report to be carried out using the two contrasting cultivars and finding the gene expression in root and shoot tissue in normal vs. stress (N-deficient) conditions. As per our knowledge only one study has been conducted in bread wheat taking flag leaf and second leaf tissue after 0 DPA and 10 DPA from three different cultivars having high, medium, and low NUE (Sultana et al., 2020).

## Materials and Methods

### Plant Materials, Nitrogen Treatments, and Plant Sampling

Plant material included two wheat (*Triticum aestivum*) cultivars, PBW677 (PFAU/MILAN/5/CHEN/*Ae. squarrosa*//BCN/3/VEE#7/BOW/4/PASTOR) known to show efficiency at a low input of nitrogen (called N efficient) and PBW703 (called N inefficient) (BWL9250*3//*Yr10*/6*Avocet/3/BW9250*3//*Yr15*/6*Avocet) with low NUE (Shamshad, 2019). The field experiments were carried out using a split-plot design with two N treatments (control and no nitrogen) as the main plot and the two cultivars in three replications as sub plots (2.4 m × 5 m). With each subplot, seeds were sown in 12 5 cm-long rows with 20 cm spacing. The nitrogen was supplied at the rate of 120 Kg ha-1 as controlled conditions and no nitrogen was supplied to induce stress conditions. The crop was grown by following the standard agronomic packaging and practices. For RNA-seq, seeds of both the varieties were surface-sterilized and grown as two sets of three replications in a growth chamber maintained at 26/24°C with relative humidity of 75%. One set of genotypes was sown in perchlorate-contaminated soil without adding any nitrogen fertilizer as N-stress (N^−^) while the other set was given nitrogen at a recommended rate of 120 kg/acre as N-control (N^+^). To the N-control set, nitrogen fertilizer was applied in the form of urea 21 days after the germination of seeds. Twenty-4 hours after application of urea, root and shoot samples were collected both from N-stress and N-control sets at the same time. Each biological replicate constituted a pool of three plants and a total of three biological replicates were used. The collected eight tissues, (N-stress root, N-control root, N-stress shoot, and N-control shoot) from both genotypes were immediately frozen in liquid nitrogen and stored at −80°C.

### Analysis of Growth, Yield, and N Parameters

Three plots per genotype and N treatment were available for the data collection. Leaf chlorophyll content was measured by randomly selecting five plants from each row using an SPAD meter, plant height was measured using scale in cm, and biological yield and grain yield were recorded as yield per plot in Kg. Grain nitrogen was estimated by using the method proposed by Eastin (2008) from the grounded grain, straw, and leaf tissue samples. The total N content was measured using the distillation and titration method (Pelican Equipment, CIT Nagar, Chennai, Tamil Nadu, India) following the manufacturer’s protocol. NUE, N utilization efficiency, and N harvest index were calculated according to Moll et al. (1982). Seed yield relative to total N accumulation in above-ground tillers was used to calculate NUtE, and NUE was determined as the NUpE and NUtE. NHI was analyzed using the ratio of total N in grains to tillers and grains.

### RNA Extraction and Reverse Transcription

Total RNA was extracted from 100 mg of root and shoot samples using Trizol Reagent (Takara kit) as per the manufacturer’s protocol. The concentration and quality of total RNA were monitored on 1.2% agarose gels as well as on a NanoDrop™ 8,000 Spectrophotometer (Thermo Scientific). Approximately 1 µg of RNA was used as a template for first-strand cDNA synthesis (Thermo Scientific Verso cDNA Kit). Oligo (dT)_18_ primers were used during reverse transcription. The cDNA was stored at −20°C.

### RNA-Seq Analysis and High-Quality Read Statistics

To obtain a global overview of the wheat transcriptome and gene activity at a nucleotide resolution, cDNA samples were sequenced by the Illumina Nextseq500 platform. The generated raw reads were submitted to the NCBI sequence read archives (SRA) with accession number PRJNA780342. The raw sequences were assessed for quality using FastQC version 0.11.2 (Andrews et al., 2015). Adaptors clipping and quality trimming of raw reads were performed using Trimmomatic v0.39 software (Bolger et al., 2014). Low quality reads with phred score <30 and read length <50 bp were removed.

### Read Alignment and Assessment to Mapping With Reference Wheat Genome

The high-quality transcriptome reads were aligned to the indexed wheat reference genome (http://www.ensembl.org/index.html) using Burrows-Wheeler Aligner (BWA) software (BWA-0.7.17) (Li and Durbin, 2009). The reference *Triticum aestivum* genome (IWGSC RefSeq v1.0) and the associated annotations (IWGSC RefSeq v1.1) were downloaded from the International Wheat Genome Sequencing Consortium (IWGSC). Indexing of the reference genome of wheat (*Triticum aestivum*) was done using BWA-0.7.17 (Li and Durbin, 2009). The resulted output sequence alignment map (SAM) files were converted into binary alignment map (BAM) format, sorted, and indexed using samtools v1.10 (Li et al., 2009).

### Differential Gene Expression Analysis

The statistical model of the Cufflinks-cuffdiff v2.2.1 package (Trapnell et al., 2012) was used to assemble and quantify differential gene expression in terms of FPKM (fragments per kilobase per million reads). Genes with log2 fold change (FC) values > 2 were considered upregulated whereas FC < −2 were considered downregulated. These genes were further categorized on the basis of statistical significance (*p* < 0.05).

### Functional Annotation and Gene Ontology Term Analysis

Functional Gene Ontology was performed with OmicsBox_windows-64_1_3_11 (http://wego.genomics.org.cn/cgi-bin/wego/index.pl). The FASTA sequences of all the upregulated and downregulated genes were downloaded from the Ensemble Plants database (https://plants.ensembl.org/index.html) and used as input into OmicsBox which contains information of all the genes assigned into three main GO domains, viz., biological process, cellular component, and molecular function. All DEGs were annotated according to wheat IWGSC release 1.1, whereas the sequences lacking annotation in the wheat reference were annotated with *Arabidopsis thaliana* (Supplementary Tables S1, S2). Volcano plots were made using R 4.0.4 (ggplot2 package) software which relates the observed differences in gene expression associated with Cuffdiff’s statistical model. Venn diagrams were constructed to represent up and downregulated DEGs using the Venny 2.1 tool (https://bioinfogp.cnb.csic.es/tools/venny/) to analyze tissue-specific and tissue-independent genes. R-based 4.0.4 software was used to make heat maps which present hierarchical clustering based on log2 fold changes to visualize the expression patterns of DEGs.

### Enrichment Analysis Based on Gene Ontology Terms

To see which class or category of genes were overrepresented and underrepresented among the differentially expressed genes in response to N stress, enrichment analysis was carried out using Fisher’s exact test with FDR <0.05. Enriched bar graphs were made using OmicsBox version 2.0.36.

### KEGG Pathway Analysis

In order to reveal the pathways to which DEGs under N-stress belong, KEGG pathway analysis was carried out by using the FASTA sequences of all the differentially expressed genes as input to the OmicsBox 2.0.36 combined pathway analysis plugin.

### Real-Time Quantitative Polymerase Chain Reaction Analysis

Real-time PCR was performed using the LightCycler 96 Roche Real-time PCR system and PowerUp™ SYBR™ Green Master Mix (applied biosystem by Thermo Fisher Scientific). Seventeen differentially expressed genes (from shoots and roots of both low N and high N) were selected for validation. Primer3 version 2.4.0 was used to design gene-specific primers and their specificity was verified using the NCBI database through the Blast tool (Supplementary Table S5). The 10 µl RT-qPCR reaction contained 1 µl of template cDNA (20 ng), 1 µl of forward primer, 1 µl of reverse primer, 4 µl of PowerUp™ SYBR™ Green Master Mix, and 3 µl of H_2_O. PCR was run at an initial denaturation of 94°C for 3 min followed by 40 cycles of 94°C for 10 s, 60°C for 30 s, 72°C for 30 s, and a final extension at 72°C for 10 min to check the specificity of amplification. The housekeeping gene TaATP (ATP-dependent 26S proteasome regulatory) (Paolacci et al., 2009) was used as the endogenous control and all reactions were performed in triplicate. Relative gene expression was analyzed using the 2−^ΔΔCt^ method.

## Results

### Plant Growth, Total Chlorophyll, and Total N Content Analysis

**FIGURE 1:**
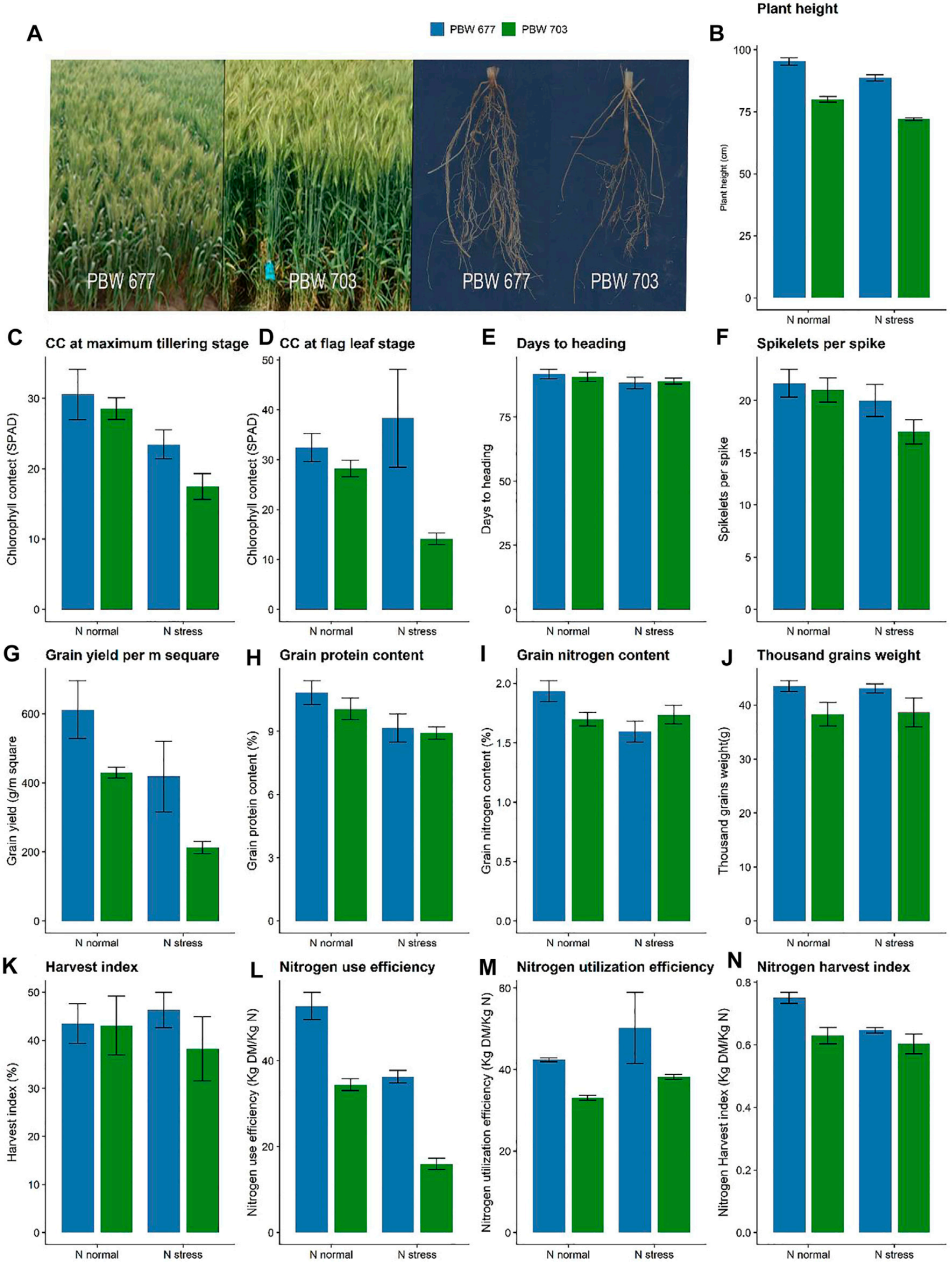
Growth performance, yield and nitrogen use efficiency parameters of wheat cultivar PBW 677 and PBW 703 grown under N^+^ and N stress conditions. **(A)** Images of plants shoot and roots **(B)** Plant height **(C)** Chlorophyll content and maximum tillering stage **(D)** Chlorophyll content at flag leaf stage **(E)** Days to heading **(F)** Spikelets per spike **(G)** Grain yield (g/m2) **(H)** Grain protein content **(I)** Grain nitrogen content **(J)** Thousand grains weight **(K)** Harvest index **(L)** Nitrogen use efficiency (NUE) **(M)** Nitrogen utilization efficiency (NUtE) **(N)** Nitrogen harvest index.

Both the cultivars PBW677 and PBW703 showed differences in growth and yield characteristics under contrasting N conditions (Figure 1A). Plant height showed no differences between the cultivars (based on cultivar) however it showed a significant difference at contrasting N levels (Figure 1B). The chlorophyll content showed a significant difference among the cultivars at different N levels (Figures 1C,D). Further no differences in the spikelets per spike were found under various N levels (Figure 1F). Grain and straw N were lower in PBW703 under N-stressed conditions in comparison to PBW677. The 1,000 grain weight was significantly higher for PBW677 under all the N fertilization regimes (Figure 1J) However, no significant difference was observed in spikelets per spike at different N levels. The nitrogen utilization and harvest index were higher in PBW677 vs. PBW703 supporting the fact that PBW677 is more efficient in acquiring, locating, and using N for grain development. Indeed, total tiller N levels as well as the NHI were significant higher in PBW677 compared to PBW703 (Figures 1M,N).

### Transcriptomics Quality and Mapping Statistics

A total of 132 Gb 150-bp paired end (PE) reads were generated through Illumina NextSeq^500^ from eight samples (2 genotype × 2 treatment × 2 tissue) including N^−^ and N^+^ treatment. On average, 18 million clean reads were obtained after trimming/clipping from each library with sizes ranging from 10 to 17 GB. The reads were of high quality at 98 and 95% and had a phred score of Q33. Moreover, the average GC% of each library was about 56% (Table 1). After mapping against the wheat reference genome (IWGSC release 1.0), varying contents of perfectly mapped reads in both roots (68.35%) and shoots/leaves (86.58%) were recorded in both genotypes (Table 2). Transcript profiles of the RNA-seq data were analyzed by calculating the read fragments per kilobase per million mapped reads (FPKM).

**TABLE 1:** Quality of transcriptomics sequenced data of root and shoot tissues of PBW677 and PBW703 genotypes.

Sample name	Direction strand	N level	Raw read no.	Clean read no.	Clean reads	GC%
Root PBW677	Forward	N+	22,427,230	21,759,104	97.02092	56
	Reverse	N+	22,427,230	21,759,104	97.02092	56
Shoot PBW677	Forward	N+	24,320,113	23,719,546	97.53057	58
	Reverse	N+	24,320,113	23,719,546	97.53057	58
Root PBW677	Forward	N-	16,938,993	16,528,622	97.57736	55
	Reverse	N-	16,938,993	16,528,622	97.57736	55
Shoot PBW677	Forward	N-	19,982,451	19,321,385	96.69177	54
	Reverse	N-	19,982,451	19,321,385	96.69177	54
Root PBW703	Forward	N+	17,137,156	16,595,928	96.84179	54
	Reverse	N+	17,137,156	16,595,928	96.84179	54
Shoot PBW703	Forward	N+	15,424,751	15,122,142	98.03816	57
	Reverse	N+	15,424,751	15,122,142	98.03816	57
Root PBW703	Forward	N-	18,294,262	17,759,653	97.07772	57
	Reverse	N-	18,294,262	17,759,653	97.07772	57
Shoot PBW703	Forward	N-	15,692,834	14,946,663	95.24515	54
	Reverse	N-	15,692,834	14,946,663	95.24515	54

**TABLE 2:** Mapping statistics of the transcriptomics data.

Cultivar	Samples	Total mapped (%)	Perfect match (%)	Unmapped (%)
PBW677	Root N+	91.72	76.00	8.2800
	Shoot N+	98.87	83.90	1.1300
	Root N-	97.26	78.12	2.7400
	Shoot N-	99.27	82.61	0.7300
PBW703	Root N+	77.30	68.35	22.7000
	Shoot N+	96.96	84.26	3.0400
	Root N-	94.66	84.57	5.3400
	Shoot N-	97.83	86.58	2.1700

### Differential Gene Expression in Response to Nitrogen Stress

**FIGURE 2:**
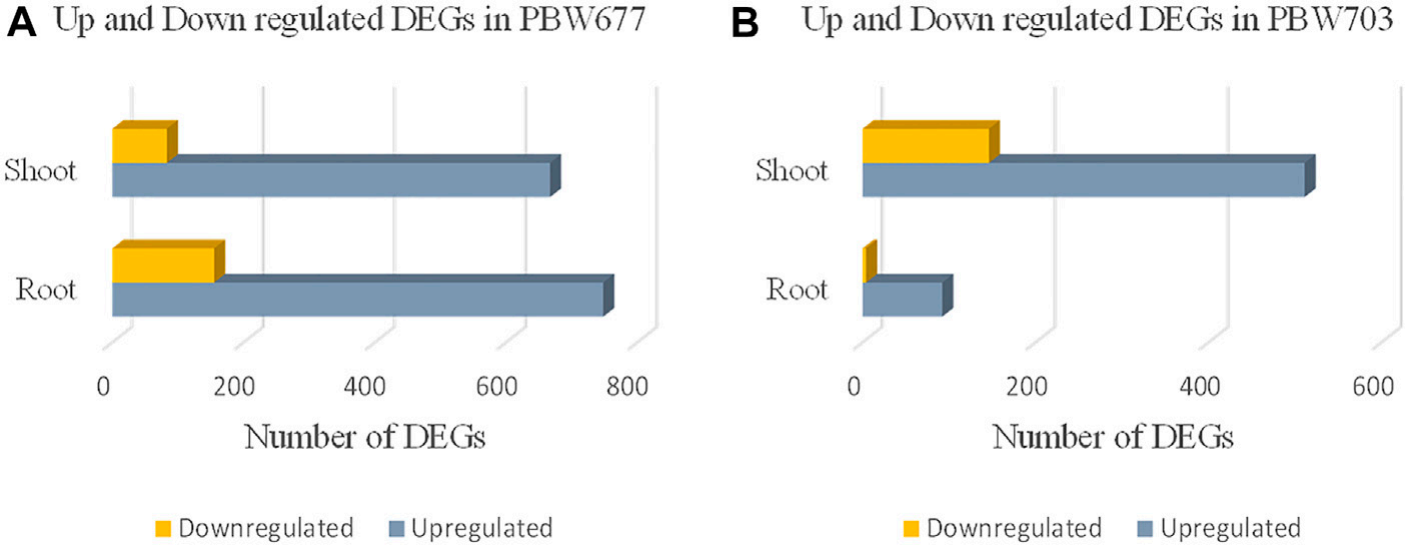
Numbers of up and downregulated DEGs (differentially expressed genes) expressed in root and shoot tissues of **(A)** PBW677 and **(B)** PBW703.

**FIGURE 3:**
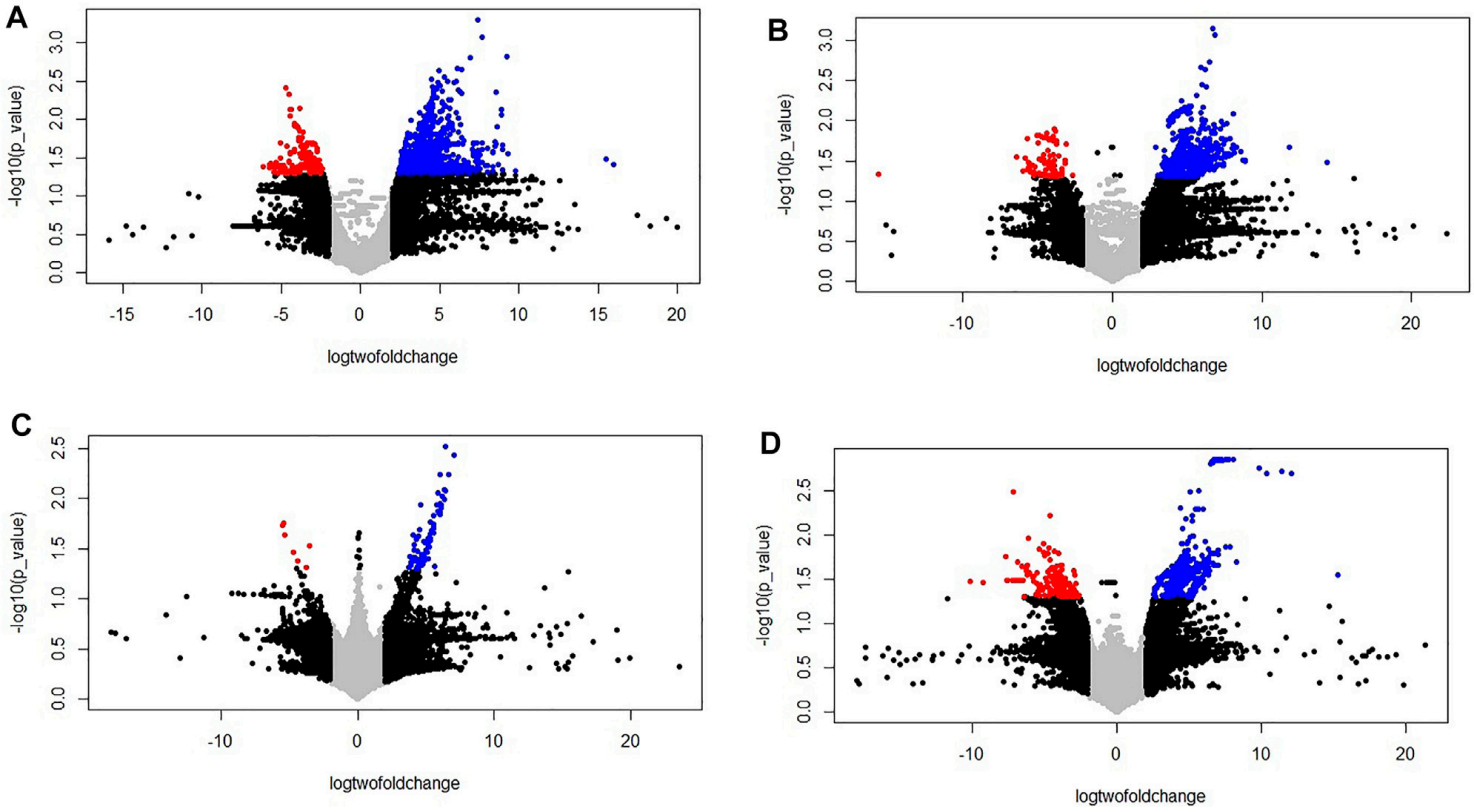
Volcano plot of the differentially expressed genes (control vs. stressed) in the two genotypes PBW677 and PBW703 for each tissue investigated in this work. **(A)** Volcano plot of gene expression in the efficient genotype in roots and **(B)** shoot/leaves; **(C)** Volcano plot of gene expression in the inefficient genotype in roots and **(D)** shoots/leaves. *The two vertical dotted lines are twice the difference threshold (<-2 or >2), and the horizontal dotted line represents significance level of -log (*p*-value = 0.05) 1.30. Red dots indicate the downregulated genes, blue dots indicate the upregulated genes, black dots represent non-significantly differentially expressed genes surpassing the threshold absolute log2 fold change (≥2) or significantly expressed genes with lower absolute log2 fold change (≤2) than the threshold, and grey dots indicate non-significantly expressed genes with lower absolute log2 fold change (≤2) than the threshold.

Transcriptomics analysis of N-efficient (PBW677) and N-inefficient (PBW703) genotypes revealed a significant difference of adaptive response in terms of gene expression pattern when there was nitrogen stress. In PBW677, the total number of genes expressed in root tissues was 70,825, out of which 903 genes showed significant differential expression (748 genes upregulated and 155 downregulated) while in shoot tissues, a total of 61,895 genes were expressed, out of which 750 were significant differentially expressed genes (667 upregulated, 83 downregulated) (Figure 2; Table 3). In contrast, the total number of expressed genes in root tissues of PBW703 was 22,046, of which only 96 were differentially expressed genes (92 upregulated and four downregulated) and in shoot tissues, 49,121 genes were expressed, out of which 657 were differentially expressed genes (511 upregulated and 146 downregulated), as shown in Figure 2. Supplementary Tables S1, S2 present the list of DEGs in root and shoot tissues of the cultivars PBW677 and PBW703, respectively. Volcano plots in Figure 3 present the most significant upregulated and downregulated genes in root and shoot tissues of both the cultivars.

**TABLE 3:** Total number of differentially expressed genes (DEGs) and number of DEGs filtered on the basis log2FC≥|2| and statistical significance (*p* < 0.05) in shoot and root tissues of PBW677 and PBW703 between high (N+)/low (N-) levels.

Comparisons	Total genes	DEGs	Upregulated Genes	Downregulated Genes
PBW677 root (N+/N-)	70,825	903	748	155
PBW677 shoot (N+/N-)	61,895	750	667	83
PBW703 root (N+/N-)	22,046	96	92	4
PBW703 shoot (N+/N-)	49,121	657	511	146

### Genes Involved in Primary Nitrogen Metabolism

**FIGURE 4:**
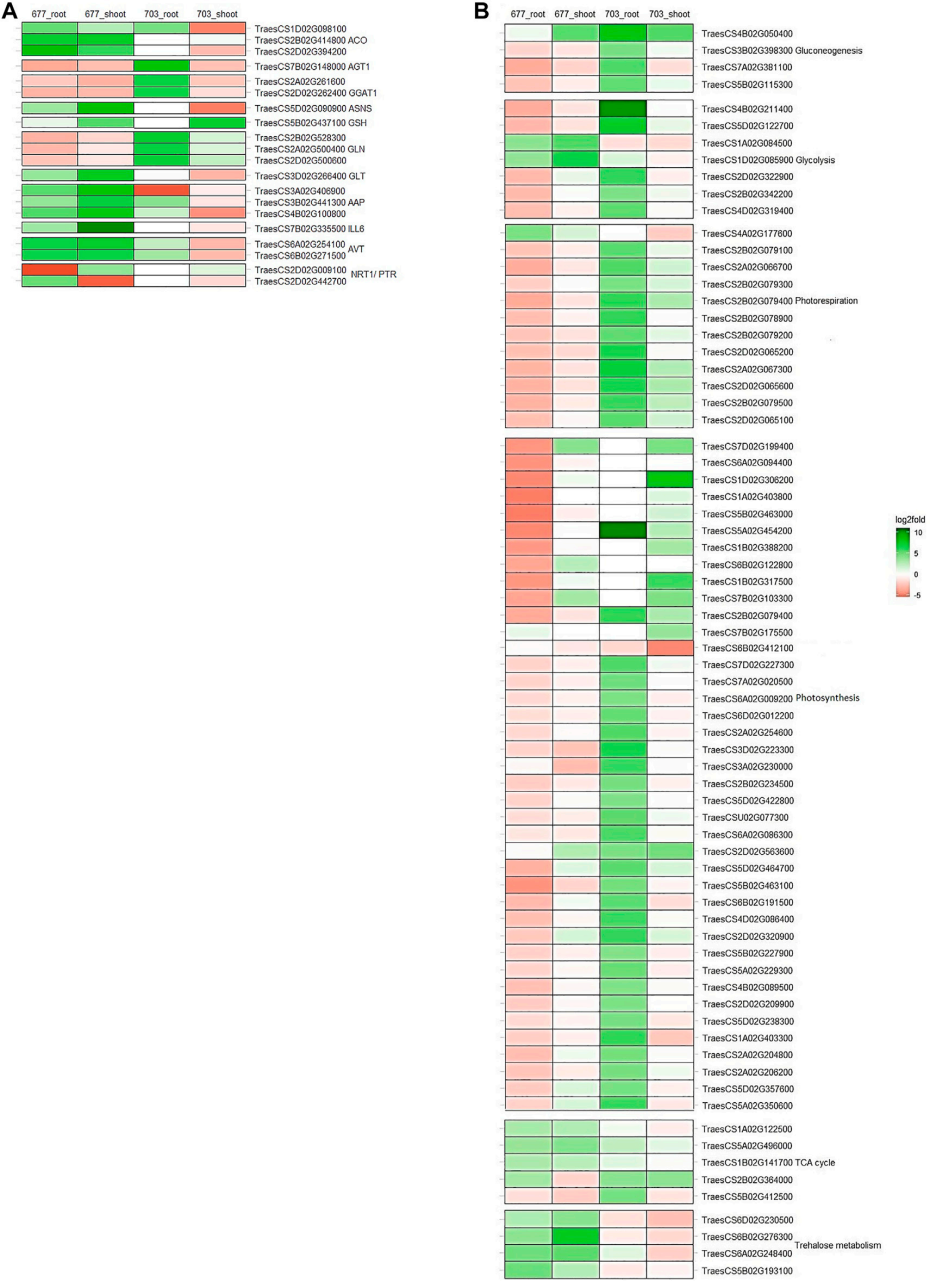
Heatmaps showing the expression patters of genes involved in between tissues and in both genotypes **(A)** primary nitrogen metabolism **(B)** carbon metabolism. *Colours indicate the differential gene expression in nitrogen stressed tissue; upregulated: green; downregulated: red; Do not have significant expression: white.

Genes involved in N absorption and assimilation were found to be differentially expressed in N stress-tolerant cultivar PBW677 compared to N stress-intolerant cultivar PBW703. Genes corresponding to the NRT1/PTR gene family were found to be upregulated in root tissues of PBW677 and downregulated in shoot tissues of PBW677 and PBW703. *Glutamate synthase* (GLT), amino acid transporter (AVT), *1-aminocyclopropane-1-carboxylate synthase* (ACO), IAA-amino acid hydrolase (ILL6), amino acid permease (AAP), and *Asparagine synthase* (ASNS) were upregulated in both the root and shoot tissues of PBW677 in comparison to PBW703 where these genes showed downregulation. However, some DEGs related to *serine--glyoxylate aminotransferase* (AGT), *glyoxylate aminotransferase* (GGAT1), and chloroplast/mitochondrial *Glutamine synthetase* (GLN) were upregulated in roots of PBW703 compared to PBW677. In addition, *Glutamate dehydrogenase* (GSH)-encoding genes were upregulated in shoot tissues of both the cultivars (Figure 4A, Supplementary Table S3).

### Carbon Metabolism Genes

Alternate gene expression was observed for genes involved in carbon metabolism, especially involved in pathways like glycolysis, tricarboxylic acid (TCA) cycle, gluconeogenesis, photorespiration, photosynthesis, and trehalose metabolism as represented in Figure 4B. It has been observed that several DEGs related to glycolysis such as glyceraldehyde-3-phosphate dehydrogenase GAPA1, fructose-bisphosphate aldolase, and fructose-1,6-bisphosphatase were downregulated in both the tissues of PBW677. In contrast, in PBW703, these genes showed upregulation in root tissues. The same pattern was observed for gluconeogenesis metabolism-related genes, such as fructose-bisphosphate aldolase, sedoheptulose-1,7-bisphosphatase, and UDP-glucose 6-dehydrogenase that were found to be downregulated in roots of PBW677 but upregulated in PBW703. Similarly, the majority of DEGs involved in photosynthesis like ribulose bisphosphate carboxylase, chlorophyll a-b binding protein of LHCII, oxygen-evolving enhancer protein 2, photosystem II 5 kDa protein, photosystem I reaction center subunit III, cytochrome b6-f complex iron-sulfur subunit, and ferredoxin-NADP reductase were upregulated in both the tissues of PBW703 (Figure 4B, Supplementary Table S3). Other than this, the resulting DEGs that were involved in energy metabolic pathways other than carbon metabolism such as genes coding NADP-dependent malic enzyme and trehalose-phosphate phosphatase were found to be strongly upregulated under N stress in both tissues of PBW677 but were considerably downregulated in PBW703.

### Transcription Factor (TF)-Encoding Genes

Transcription factors play a vital role as molecular switches controlling the expression of specific genes and play crucial roles in plant development, cell cycling, cell signaling, and stress responses. Due to N stress, various unique genes encoding transcription factors were differentially expressed in N stress-tolerant cultivar PBW677 corresponding to different TF families of MYB, WRKY, RING finger protein, Zinc finger protein, TIFY proteins, AP2/ERF, HSP, BTB/POZ domain, NDR1/HIN1domain, bZIP, GATA, bHLH, DIVARICATA factors, Bowman-Birk type trypsin inhibitor isoform, BAG family molecular chaperone, dnaJ protein, ARF, and other families (Supplementary Table S3). In contrast, TF-encoding genes were identified in N stress-sensitive cultivar PBW703 and the number of TF-related DEGs in each family was less than those found in PBW677. We also observed the tissue-specific expression of several TF families. For instance, most of WRKY, HSP and AP2/ERF genes were extensively upregulated in roots only, however genes related to the BAG family molecular chaperone, ARF, DIVARICATA, GATA, and dnaJ protein were upregulated majorly in shoots of PBW703. Additionally, MYB and bHLH genes were expressed in both the tissues of PBW677 (Supplementary Fig. S1, Supplementary Table S3).

### Transporter Encoding Genes

Genes encoding for various transporters responsible for uptake and transport of nutrients were found to be differentially expressed under N stress in both the cultivars. In general, it was observed that the genes belonging to different transporter families were shown to be unregulated in both the tissues of PBW677 in comparison to PBW703 where they were downregulated. In particular, calcium-binding protein transporter genes were upregulated in both the tissues of PBW677 in addition to the ABC transporter and SWEET transporter (Supplementary Fig. S2, Supplementary Table S3).

### Protein Kinases Encoding Genes

In this study, various unique protein kinases were differentially expressed in both the tissues. The identified PK genes belonged to classes CDK (cyclin-dependrnt kinase), CIPK (CBL-interacting protein kinase), cysteine-rich domains, F-box domains, LRR (leucine rich repeats), MAPK (mitogen activated protein kinase), serine threonine protein kinase, serine arginine protein kinase, and U-box domains (Supplementary Fig. S3, Supplementary Table S3). We found PKs belonging to MAPK, serine threonine protein kinase, CDK, CIPK, and cysteine-rich domains upregulated in roots of PBW677. Besides, most members of F-box domains and LRR were found upregulated only in shoots of PBW677.

### Other Stress-Related Genes

**FIGURE 5:**
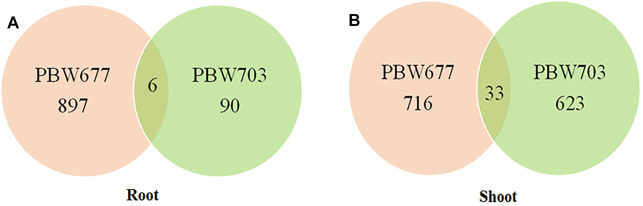
Common DEGs (differentially expressed genes) in root and shoot tissues of **(A)** PBW677 and **(B)** PBW703.

In this study, we also observed upregulation of a number of genes related to detoxification and protection from oxidative stress, the majority of which were found in both tissues in N stress-tolerant cultivar PBW677. These mainly belong to classes of glutathione S-transferases (GSTs), cytochrome P450 (CYP450), E3 ubiquitin-protein ligase, peroxidase and phenylalanine ammonia-lyase, and aquaporin PIP1-1. Most of the genes related to glutathione S-transferases (GSTs), phenylalanine ammonia-lyase (PAL), and E3 ubiquitin-protein ligase were upregulated in both root and shoot tissues of PBW677 (Figure 5) (Supplementary Figure S4, Supplementary Table S3).

### Common Genes Between Root and Shoot Tissues of PBW677 and PBW703

Among the total DEGs studied, common genes showing up and downregulation were detected, also the genes showing alternate expression (that is upregulation in one cultivar and downregulation in another cultivar) were identified. A list of common genes with their log fold change value and involved biological pathways is represented in Supplementary Table S4.

### Enriched Gene Ontology Terms in Differentially Expressed Genes Under Nitrogen Stress

**FIGURE 6:**
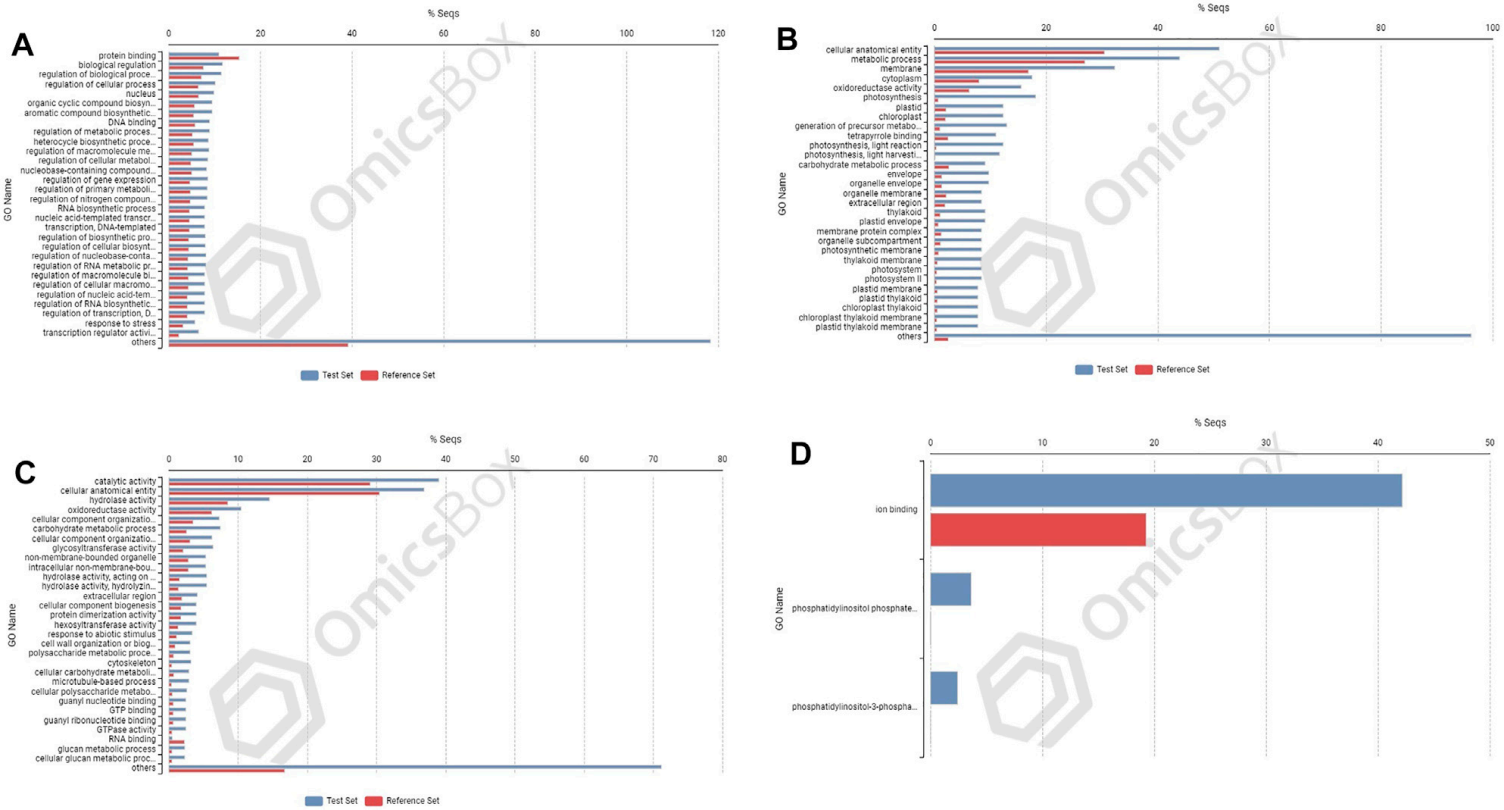
Enrichment analysis showing genes present in roots of PBW677: **(A)** upregulated and **(B)** downregulated; and in shoot tissues: **(C)** upregulated and **(D)** downregulated.

**FIGURE 7:**
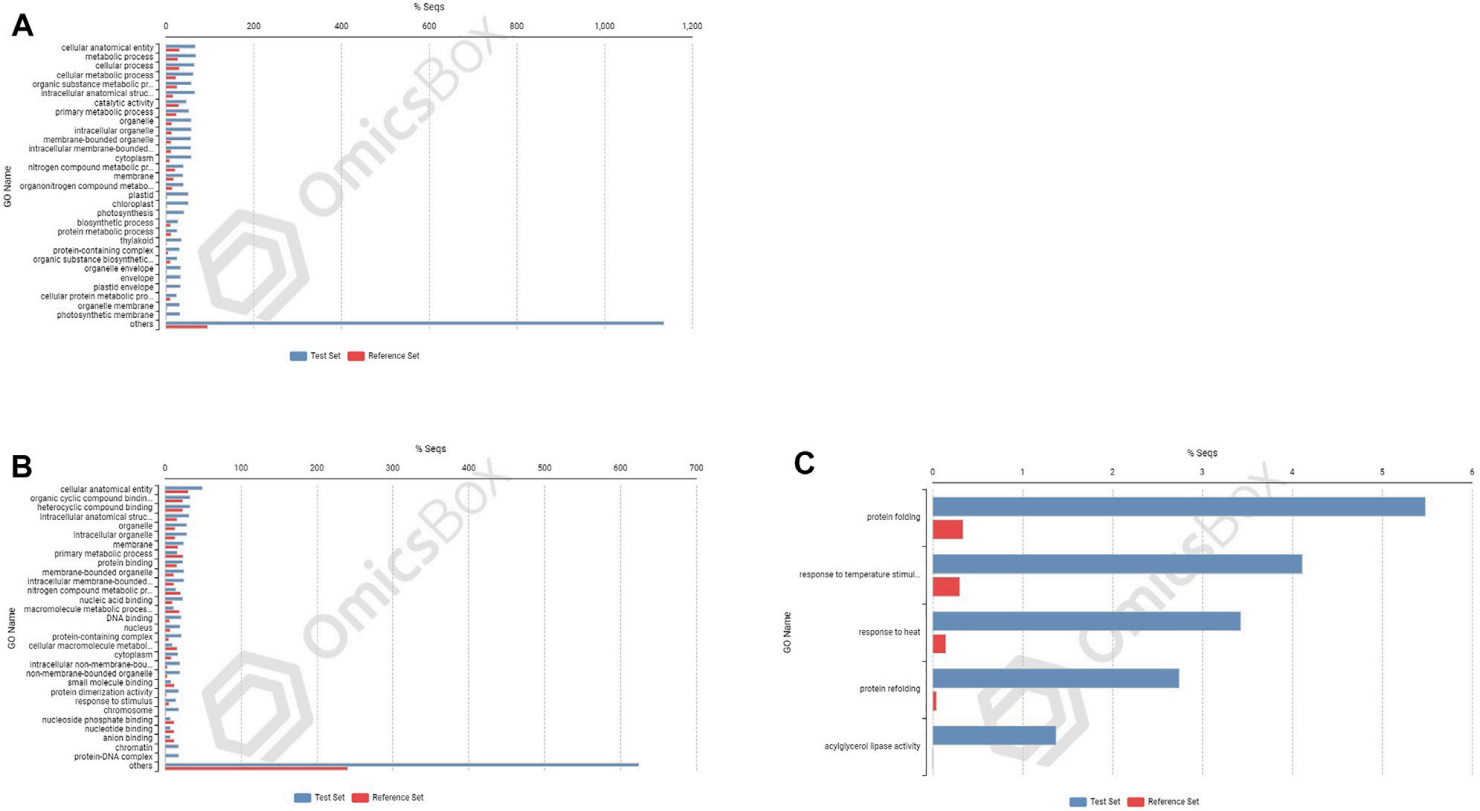
Enrichment analysis showing genes present in roots of 703 **(A)** upregulated genes; and in shoot tissues: **(B)** upregulated and **(C)** downregulated.

In cultivar PBW677, enrichment analysis using Fisher’s exact test depicted the over and underrepresented genes expressed in response to nitrogen stress, as shown in Figure 6. Among the upregulated DEGs in root tissues of cultivar PBW677, 140 genes belonging to different GO categories (biological process, cellular component, and molecular function) were found to be overrepresented. Most of the overrepresented class of genes was found to be involved in sequence-specific DNA binding, response to temperature stimulus, abiotic stimulus, and transcription factors involved in transcription regulation and defense responses. Among the underrepresented class, only 11 genes were found, most of which belonged to the process of translation. The top 10 biological processes that were overrepresented among the upregulated DEGs expressed in root tissues of PBW677 are represented in Table 4. Among the downregulated DEGs in root tissues of PBW677, there were 64 genes which were overrepresented and belonged to the biological processes photosynthesis, carbon fixation, and photorespiration, as represented in Table 5. However, in the shoot tissues of PBW677, 82 upregulated genes were found to be overrepresented and six were underrepresented. In contrast, among the upregulated DEGs in root tissues of PBW703, 163 genes were found to be overrepresented and among them the biological processes photosynthesis, phosphorespiration, carbon fixation, and carbon metabolism, glutamine biosynthetic process, and nitrogen compound metabolic processes were highly enriched and overrepresented, as represented in Figure 7. In the shoot tissues of PBW703, among the upregulated DEGs (Figure 7), the overrepresented biological processes included nucleosome assembly, chromatin assembly, DNA packaging, chromatin remodeling, chromosome organization, response to inorganic substance, abiotic stimulus, lipid transport, sulfur compound metabolic process, glutathione metabolic process, nitrate transport, and response to nitrate with a number of 126 genes. Table 6 represents the top 10 highly enriched biological processes overrepresented in upregulated DEGs in shoot tissues of PBW703. The underrepresented category included macromolecule modification, protein metabolic process, protein modification process, and nitrogen compound metabolic process. Protein folding and refolding and responses to heat and temperature stimulus were among the highly enriched biological processes among downregulated DEGs in shoot tissues of PBW703.

**TABLE 4:** List of top 10 biological processes with GO IDs that are overrepresented in upregulated DEGs in root tissues of PBW677.

S. No.	GO name	GO IDs	FDR
1	Response to temperature stimulus	GO:0009266	1.088379E-10
2	Response to abiotic stimulus	GO:0009628	5.055289E-8
3	Response to osmotic stress	GO:0006970	1.245807E-7
4	Response to heat	GO:0009408	1.245807E-7
5	Phosphoenol pyruvate family amino acid metabolic process	GO:1902222	1.684675E-7
6	Phenylalanine catabolic process	GO:0006559	1.684675E-7
7	Response to salt stress	GO:0009651	1.684675E-7
8	Aromatic amino acid family catabolic process	GO:0009074	3.512503E-6
9	Defense response to bacterium	GO:0042742	5.271174E-5
10	Secondary metabolite synthesis	GO:0044550	2.678367E-4

**TABLE 5:** List of top 10 biological processes with GO IDs that are overrepresented in downregulated DEGs in root tissues of PBW677.

S. No.	GO name	GO IDs	FDR
1	Photosynthesis	GO:0015979	1.08140E-50
2	Protein chromophore linkage	GO:0018298	3.63567E-33
3	Photosynthesis, light harvesting	GO:0009765	1.86844E-32
4	Photorespiration	GO:0009853	1.464116E-28
5	Photosynthesis, light reaction	GO:0019684	2.379313E-23
6	Cellular metabolic compound salvage	GO:0043094	1.711553E-22
7	Photosynthesis dark reaction	GO:0019685	1.962272E-20
8	Reductive pentose phosphate cycle	GO:0019253	1.962272E-20
9	Response to abiotic stimulus	GO:0009628	8.835918E-12
10	Glutamine biosynthetic process	GO:0006542	2.613054E-3

**TABLE 6:** List of top 10 biological processes with GO IDs that are overrepresented in upregulated DEGs in shoot tissues of PBW703.

S. No.	GO name	GO IDs	FDR
1	Nucleosome organization	GO:0034728	8.368916E-56
2	Response to stress	GO:0006950	9.207505E-18
3	Lipid transport	GO:0006869	2.889702E-7
4	Photorespiration	GO:0009853	1.464116E-28
5	Photosynthesis, light reaction	GO:0019684	2.379313E-23
6	Cellular metabolic compound salvage	GO:0043094	1.711553E-22
7	Photosynthesis dark reaction	GO:0019685	1.962272E-20
8	Reductive pentose phosphate cycle	GO:0019253	1.962272E-20
9	Response to abiotic stimulus	GO:0009628	8.835918E-12
10	Glutamine biosynthetic process	GO:0006542	2.613054E-3

### Validation of DEG Genes Using Real-Time Quantitative Polymerase Chain Reaction Analysis

**FIGURE 8:**
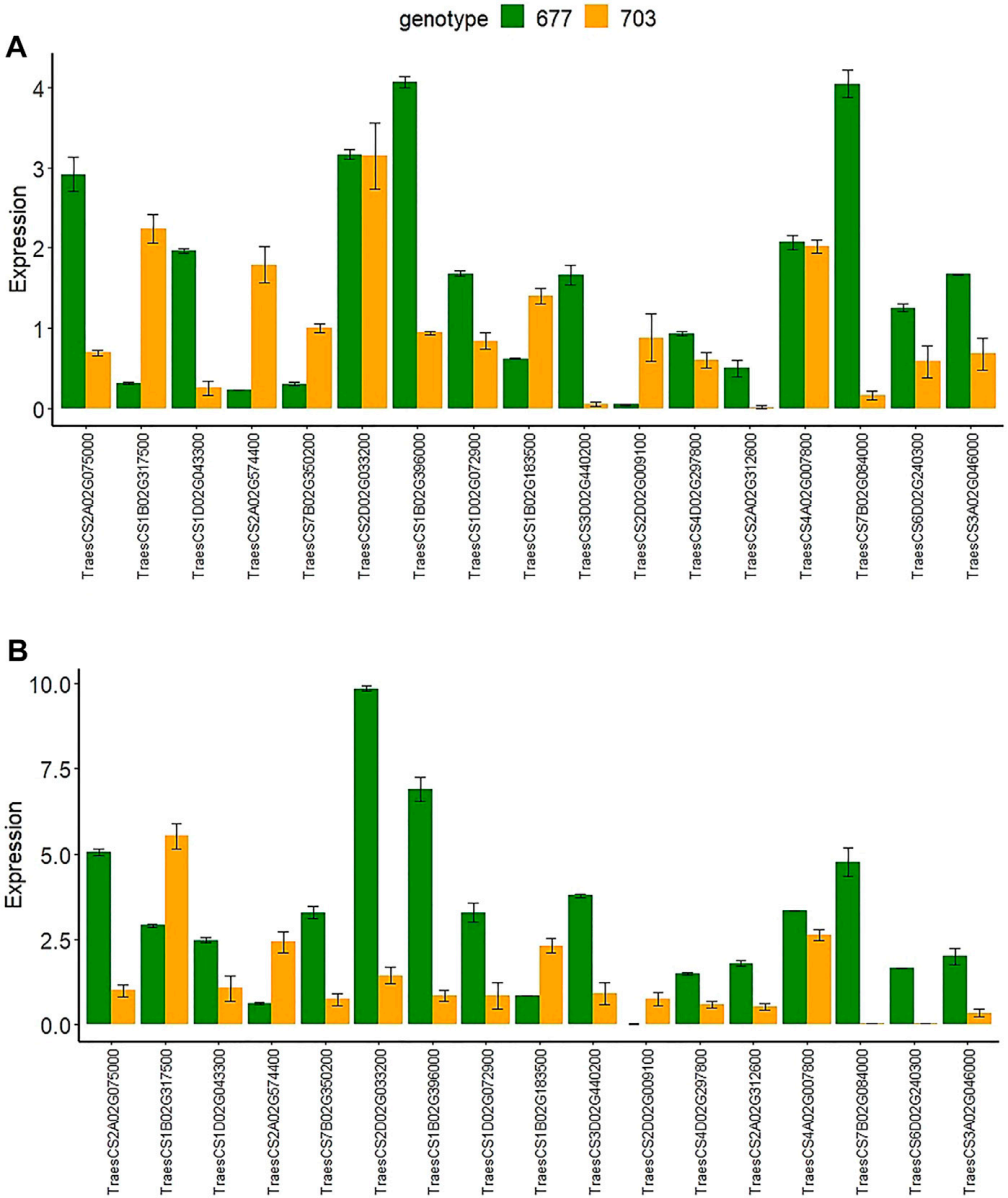
The relative gene expression of 17 randomly selected genes examined by RT-qPCR in both genotypes PBW677 and PBW703 in **(A)** roots and **(B)** shoots.

To validate the expression data obtained by RNA-seq, we performed RT-qPCR analysis of the expression of the 17 selected DEGs (Supplementary Table S5) with both upregulated and downregulated expression in both of the tissues and in both genotypes. Results were found in agreement with the RNA-seq-based gene expression pattern with minor variations in the log2 FC values (Figure 8).

## Discussion

To find out the underlying genes involved in NUE pathways, a transcriptomics study was conducted in control and N stress conditions in root and shoot tissues of two cultivars PBW677 and PBW703. Differential gene expression revealed the key genes involved in photosynthesis, starch and sugar metabolism, and nitrogen metabolism in both the cultivars. In limited N conditions, N primarily affected plant morphology, accelerating plant flowering time, senesces, decreasing the height, and lowering biomass and harvest index. The overall stresses of the plant were evident from the spikelets per spike while the average seed test grain weight was comparable. This indicated the plants concentrated their energy to produced viable seeds. The nitrogen use efficiency was affected by supplied N levels. The increase of NUE was affected by N regimes in winter and spring wheat (Cormier et al., 2013; Nehe et al., 2018). Under nitrogen stress, the number of upregulated as well as downregulated genes was higher in roots as compared to shoots. This is in accordance with the previous study conducted by Curci et al. (2017) on durum wheat as roots are primary organs responsive to N stress. Also, it was found that the number of DEGs was higher in PBW677 as compared to PBW703. This might be due to the fact that PBW677 responds actively in N-deficient conditions compared to PBW703 as in the case reported by Sultana et al., in 2020 where medium NUE Spitfire had more DEGs in comparison to low NUE cultivar Volcani. The 97 common genes between root and shoot tissues were mostly related to plant hormone signal transduction, MAPK signaling pathway, starch and sucrose metabolism, glutathione metabolism, and chlorophyll and porphyrin metabolism. The 33 DEGs common in shoot tissues were mainly involved in lipid transport, amino acid metabolism, photosynthesis, carotenoid biosynthesis, starch and sucrose metabolism, and nucleotide metabolism. Several previous studies indicated that when plants were subjected to nitrogen stress they exhibited a wide range of responses including molecular expression and biochemical processes involving various genes and pathways. There is a strong relation between nitrogen and carbon metabolism as has been shown in various studies (Duan et al., 2018; Naliwajski and Skłodowska, 2018). Gene enrichment analysis based on Gene Ontology is the method by which key biological processes responsible for stress adaptation can be revealed, for example an N deficiency study in durum wheat reported photosynthesis, carbon metabolism, and nitrogen metabolism as the highly enriched biological processes (Curci et al., 2017). Under N limitation, the metabolic process and oxidation–reduction process in wheat seedlings were enriched significantly (Wang et al., 2019). Under nitrogen stress, catalytic activity, binding, and the metabolic and cellular process were highly enriched GO terms in potato roots, shoots, and stolons (Tiwari et al., 2020). This study revealed that upregulated DEGs in root tissues of PBW677 were involved in pathways like DNA binding, response to abiotic stimulus, and defense response while the downregulated genes were involved in biological processes like photosynthesis, photorespiration, and carbon fixation. While in shoot tissues, the highly enriched biological processes were carbohydrate metabolic process, cell wall biogenesis, and cellular polysaccharide metabolic process. In contrast to this, the upregulated DEGs in root tissues of PBW703 belonged to enriched biological processes like photosynthesis, carbon metabolism, glutamine biosynthetic process, and nitrogen compound metabolic processes while in shoot tissues, the enriched pathways were involved in nucleosome assembly, sulfur compound metabolic process, glutathione metabolic process, nitrate transport, etc. The reason behind the upregulated transcripts belonging to the pathways related to nucleosome assembly and chromatin packaging would be the mechanisms involved towards the stability of the epigenome under N stress in PBW703 (Sudan et al., 2018). N metabolism-related genes were identified in the annotated transcriptome data showing both up as well as downregulation. For example expression of low affinity nitrate transporter NRT1 was found to be upregulated in root tissues of PBW677 and downregulated in shoot tissues of PBW677 and PBW703 under N stress which usually increases the expression of transport systems for nitrate and ammonium (Crawford and Glass, 1998). In bread wheat under N stress, a high level of expression of the NRT1/PTR family was found (Sultana et al., 2020). Also, it was shown that in the low N-treated plants, downregulation of the NRT1/PTR family was observed which is related to low grain yield and grain protein content (Léran et al., 2015). Other genes playing a role in N absorption, assimilation, and remobilization like glutamate synthase (GLT), amino acid transporter (AAP), and asparagine synthase were upregulated in both the root and shoot tissues of PBW677 where as these genes were downregulated in PBW703. The upregulated expression of these genes can be related to the high NUE of PBW677 which is better adapted to N stress. Aminotransferases like serine glyoxylate aminotransferase (AGT), glyoxylate aminotransferase (GGAT1), and chloroplast glutamine synthetase (GLN) were upregulated in roots of PBW703 which is supported by previous reports conducted in sorghum where a transcriptomics study found more N assimilator genes in low NUE cultivars (Singh et al., 1973).

Transcription factors (TFs) are important key regulators that play a significant role in adaptation under environmental stresses (Shahzad et al., 2020). In our study we found a number of transcription factor families up and downregulated under N stress. The number of expressed TFs was higher in PBW677 than PBW703 which might be due to the fact that effective regulatory mechanisms exist under N stress in high NUE cultivar PBW677. Several TF families that were identified in response to N stress were HSF, MYB, WRKY, and ZINC finger. Transcriptomics analysis in durum wheat identified most of the WRKY family in response to N chronic stress in roots (Curci et al., 2017). This family is one of the largest families of plant-specific transcription factors that plays important roles in various abiotic stresses (drought, saline, alkali, temperature, and ultraviolet radiation (Li et al., 2020). Several MYB and bHLH TFs were reported to be involved in regulation of target genes under plant stress (Pireyre and Burow, 2015). In rice, expression of OSMYB48-1 was reported to be upregulated in tolerance toward abiotic stress probably via the regulation of stress-mediated ABA biosynthesis (Xiong et al., 2014).

Zinc finger proteins (ZEPs) enhance tolerance under abiotic stress (Zang et al., 2016). A high level of expression was observed for protein kinases in high NUE PBW677 (either up or downregulated). Protein kinases are known to play an important role in signal transduction and are regarded to be the central regulatory components to major environmental stresses such as drought, heat, cold, and pathogen attack (Wang et al., 2020). PKs play a role in hormone signaling, cell cycle growth, and nutrient signaling as well.

Nitrogen of the plant is invested in making protein and chlorophyll content of photosynthetic apparatus thus carbon metabolism and nitrogen metabolism are interconnected and carbon metabolism is dependent on nitrogen assimilation in plants (Foyer et al., 2001). In our study we found that a number of carbon metabolism genes were downregulated like genes involved in glycolysis, TCA cycle, and gluconeogenesis. This is similar to previous reports in which it was shown that N stress negatively effects plant carbohydrate metabolism (Rufty et al., 1988; Sultana et al., 2020). We found other stress-related genes to be highly expressed under N stress in high NUE cultivar PBW677 like GSTs, cytochrome P 450, E3 protein ligase, etc. Glutathione S transferases prevent cells from oxidative damage by quenching reactive molecules with the addition of glutathione (GSH) (Kumar and Trivedi, 2018).

## Conclusion

RNA sequencing of two contrasting cultivars PBW677 and PBW703 for NUE helped in revealing candidate genes which could be utilized in future breeding programs focused on reducing the use of nitrogen fertilizers. The majority of the genes belonged to transcription factor families; protein kinases and stress-related nitrogen metabolism were found to be highly expressive in wheat cultivar PBW677 which might explain its behavior under N stress. There was a difference in highly enriched pathways responsive to nitrogen stress in the contrasting cultivars which might be the cause for their different behavior towards N stress. The 13 common genes showed alternative expression patterns in PBW677 and PBW703 and could be the potential candidates for high NUE-targeted breeding.

## Data Availability

The datasets presented in this study can be found in online repositories. The RNA-seq data have been deposited in the NCBI. Submission details: NCBI Sequence Read Archive (SRA) submission: PRJNA780342.
