# Supplementary material for: RNA-Seq-Based Transcriptomics Study to Investigate the Genes Governing Nitrogen Use Efficiency in Indian Wheat Cultivars
Source: Front Genet. 2022 Mar 31;13:853910. doi: 10.3389/fgene.2022.853910 (PMC9009086; doi:10.3389/fgene.2022.853910)
Supplement: Supplementary file 3 [file DataSheet1.pdf]

RNA seq based transcriptomics study to investigate the genes governing nitrogen use efficiency in Indian wheat cultivars

Sarabjit Kaur[1†], M. Shamshad[2†], Suruchi Jindal[1], Amandeep Kaur[1], Satinder Singh[2], Achla sharma[2*] and Satinder Kaur[1*]

1. School of Agricultural Biotechnology, Punjab Agricultural University, Ludhiana, India
2. Department of Plant Breeding and Genetics, Punjab Agricultural University, Ludhiana, India

†Sarabjit Kaur, M. Shamshad contributed equally to this work.

*Corresponding author email: satinder.biotech@pau.edu, achla@pau.edu

SK: 0000-0003-0672-9268
AK: 0000-0002-9140-3424
SK: 0000-0003-3704-3074

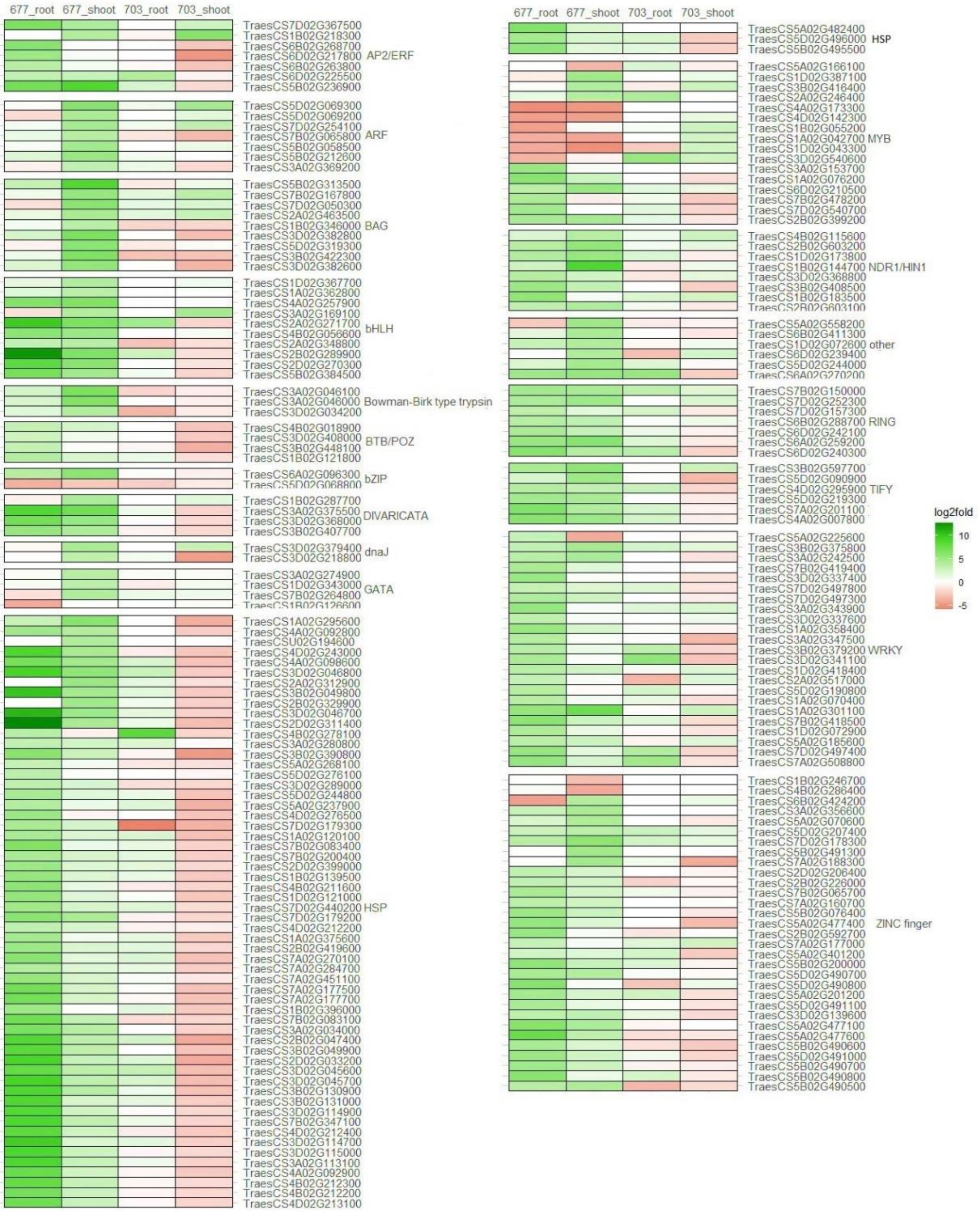

Supplementary Figure 1. Heatmap showing the expression patters of genes involved as trancription factors response in between tissues and also in both genotypes.
* Colours indicate the differential gene expression in nitrogen stressed tissue; upregulated, green, downregulated, red, white: not differentially expressed.

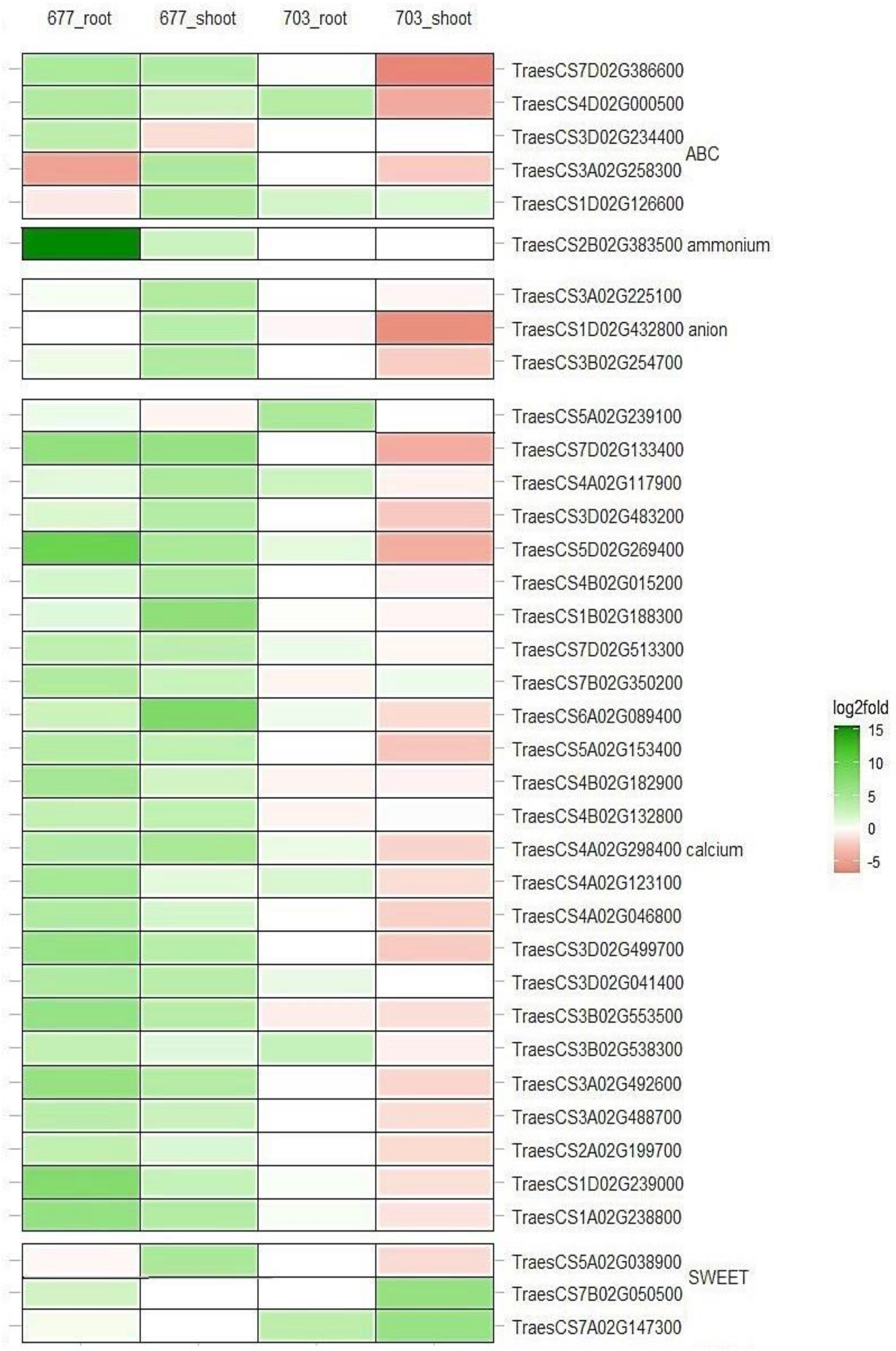

Supplementary Figure 2. Heatmap showing the expression patters of genes involved as transporter in between tissues and also in both genotypes.

* Colours indicate the differential gene expression in nitrogen stressed tissue; upregulated, green, downregulated, red, white: not differentially expressed.

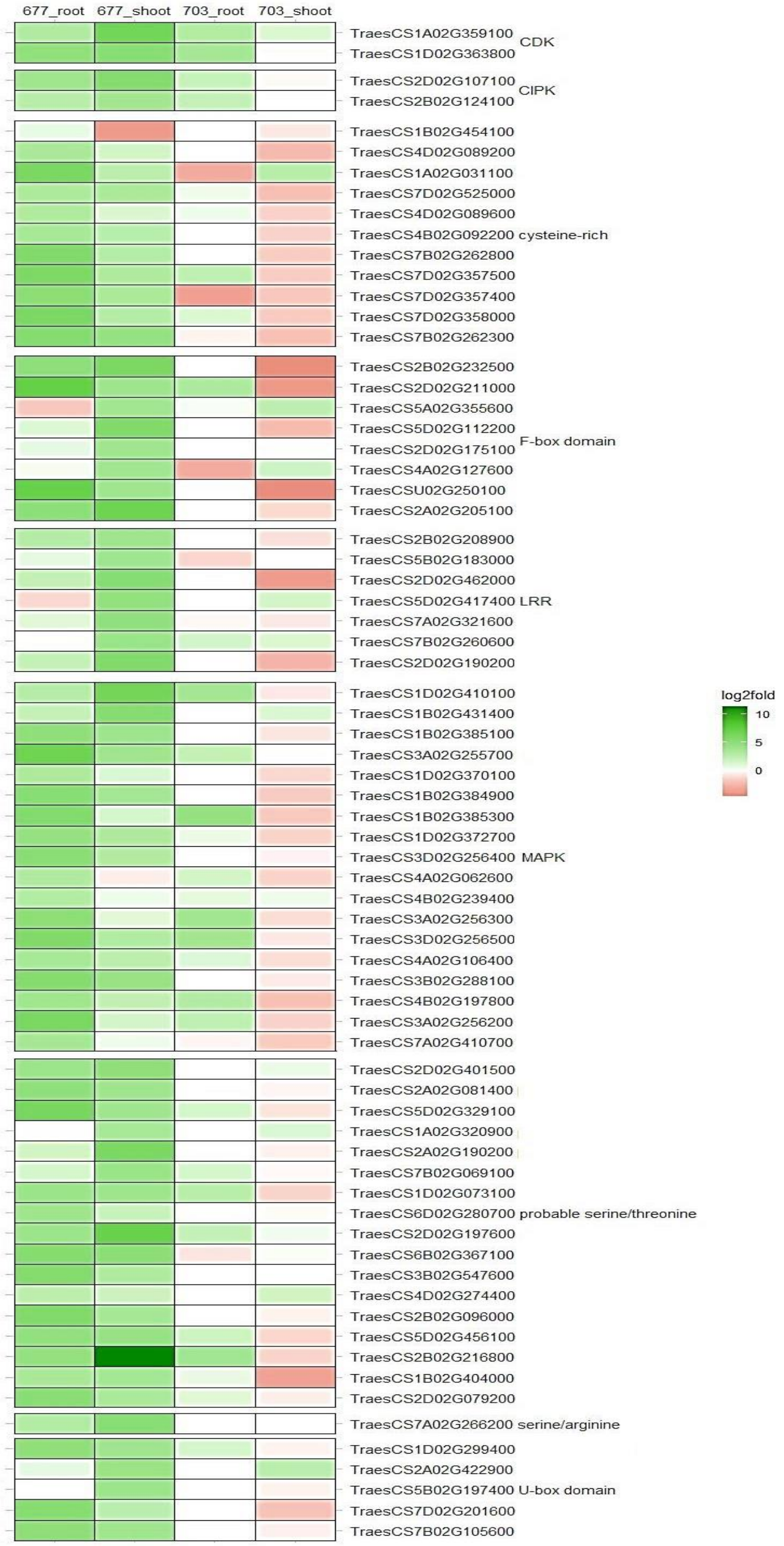

Supplementary Figure 3. Heatmap showing the expression patters of genes involved as protein kinases in between tissues and also in both genotypes.

* Colours indicate the differential gene expression in nitrogen stressed tissue; upregulated, green, downregulated, red, white: not differentially expressed.

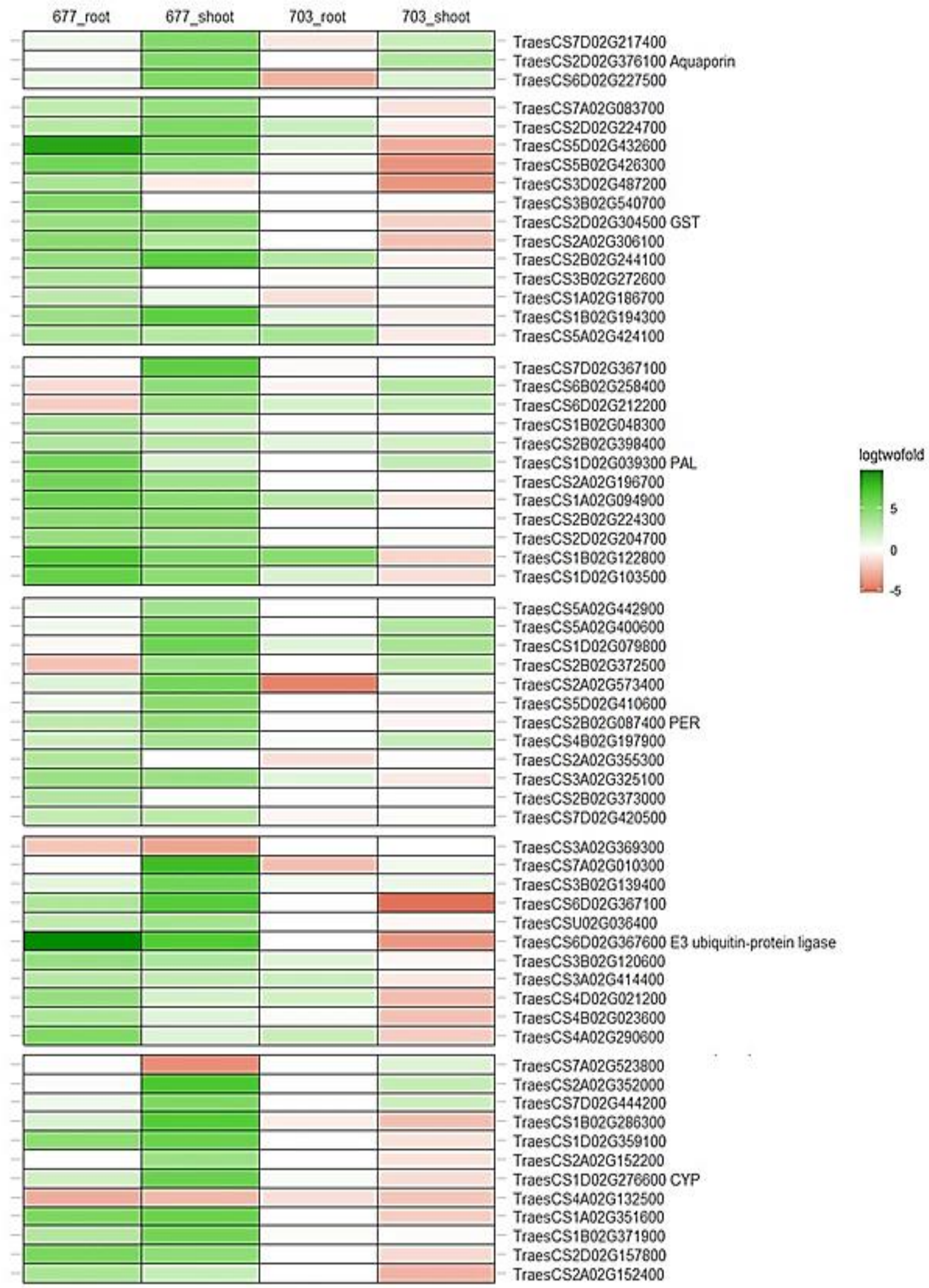

Supplementary Figure 4. Heatmap showing the expression patters of other genes in between tissues and also in both genotypes

* Colours indicate the differential gene expression in nitrogen stressed tissue; upregulated, green, downregulated, red, white: not differentially expressed.
